# Neuropathic pain characteristics in patients with pain-related temporomandibular disorders

**DOI:** 10.22514/jofph.2024.016

**Published:** 2024-06-12

**Authors:** Jeanne H.M. Baggen, Michail Koutris, Frank Lobbezoo

**Affiliations:** Department of Orofacial Pain & Dysfunction, Academic Centre for Dentistry Amsterdam (ACTA), 1081 LA Amsterdam, The Netherlands

**Keywords:** Orofacial pain, Diagnostic criteria for temporomandibular disorders, Pain-related temporomandibular disorders, Douleur neuropathique 4, Neuropathic pain

## Abstract

In orofacial pain patients, pain-related temporomandibular disorders (TMD) and neuropathic pain (NP) can both be present. The aim of this cross-sectional study was to examine whether in patients with orofacial pain, associations can be found between (subdiagnoses of) pain-related TMD and NP. Participants were asked to fill in the questionnaires of the Diagnostic Criteria for TMD (DC/TMD) and a screening questionnaire for NP, the Douleur Neuropathique 4 (DN4). Complete data sets were collected from 355 participants with an orofacial pain complaint. First, univariate analyses were used to pre-screen the independent variables. Subsequently, multivariate binary logistic regression analysis was used to further assess the association between the independent variables, which were significant in the univariate analyses, and the dependent variable NP. From all 355 participants, 274 (77.2%) had pain-related TMD. 72 participants (20.3%) had a DN4 score ≥4, suggesting the presence of NP. A DN4 score ≥4 occurred in 62 (22.6%) of the 274 cases with pain-related TMD. In the univariate analyses, NP was found to be significantly associated with the presence of pain-related TMD (χ^2^ = 4.088, *p* = 0.043), myalgia (χ^2^ = 6.916, *p* = 0.009), and headache attributed to TMD (χ^2^ = 13.366, *p *< 0.001). In the multivariate analysis, NP was only significantly associated with headache attributed to TMD (Odds Ratio = 2.37, 95% Confidence Interval: 1.30 to 4.34, *p* = 0.005). NP characteristics are associated with headache attributed to TMD. The results stress the need for including a NP assessment in diagnostic protocols for pain-related TMD.

## 1. Introduction

Orofacial pain is defined as pain in the soft and hard tissues of the face, head and neck [1]. It is caused by diseases or disorders of regional structures, by dysfunction of the nervous system, or through referral from distant sources [2]. In 2020, the International Classification of Orofacial Pain (ICOP) created a classification guide for orofacial pain [3]. This classification proposed that chronic temporomandibular disorders (TMD) may be classified as a chronic primary orofacial pain [4]. Many patients have orofacial pain complaints for several years, however without receiving causal treatment but rather more symptomatic-based management [1, 5]. Prevalence of orofacial pain is approximately 13% in the general population (range 1–48%) [6]. The most common cause of orofacial pain, after dental pain, is pain of musculoskeletal origin described under the umbrella term “pain-related TMD” [7]. The prevalence of pain-related TMD is estimated at 15% in women and 8% in men [8]. The etiology of pain-related TMD is multifactorial, whereby an imbalance between the load and the resistance of the soft and hard tissues of the masticatory system, due to oral habits, facial trauma, systemic disorders, genetic factors and psychosocial factors, can be involved. Chronic TMD could be considered as a condition with chronic primary orofacial pain, presenting as myofascial TMD pain [9]. Individuals with chronic pain often report comorbid and complex psychosocial symptoms [10]. Internationally accepted criteria for setting the diagnosis of TMD have been developed for application in clinical practice [11]. Pain-related TMD is considered nociceptive pain, thus pain “that arises from actual or threatened damage to non-neural tissue and is due to activation of nociceptors” [12].

Besides pain-related TMD, another possible non-dental cause of orofacial pain is neuropathic pain (NP). NP used to be defined as pain initiated or caused by a primary lesion or dysfunction in the peripheral or central nervous system [13]. A recent study proposed defining NP as pain arising as a direct consequence of a lesion or disease affecting the somatosensory system [13]. The Neuropathic Pain Special Interest Group (NeuPSIG) of the International Association for the Study of Pain (IASP) recently updated a grading system to assist with determining the level of certainty that a patient’s pain is actually NP. They classify NP as unlikely, possible, probable or definite, depending on the history of the complaints of the patient, the findings from a clinical examination, and possible laboratory tests [13, 14]. NP can arise either spontaneously or after the provocation of various stimuli [15]. Many screening tools for NP are available in clinical practice. These screening tools are often used to identify NP in addition to a clinical examination. The most commonly used screening tool is the “Douleur Neuropathique 4” (DN4) questionnaire [16]. This screening instrument is based on key signs and symptoms, such as burning, painful cold, electric shocks and itching [16]. To establish a more definite diagnosis of NP, a thorough diagnostic examination, including qualitative and quantitative testing of the somatosensory function, is needed [17].

It can be challenging to set a proper pain diagnosis and to distinguish between nociceptive pain and NP in patients with orofacial pain complaints. It is possible that nociceptive pain and NP merely occur simultaneously as comorbid conditions, but they may as well influence each other. An association has been found between chronic TMD pain and secondary mechanical hyperalgesia, induced experimentally outside the trigeminal area [18]. Several studies have noted that characteristics of chronic pain and NP seem to occur together [19, 20], but as yet it is not known whether pain-related TMD and NP are interrelated [21]. Therefore, the aim of this study was to examine whether in patients with orofacial pain, an association can be found between (subdiagnoses of) pain-related TMD and NP.

## 2. Materials and methods

### 2.1 Design and participants

Data were collected cross-sectionally from participants who were referred to the Clinic for Orofacial Pain and Dysfunction of the Academic Centre for Dentistry Amsterdam (ACTA) from October 2013 until November 2015. All patients were asked to fill in a questionnaire of the Diagnostic Criteria for Temporomandibular Disorders (DC/TMD) and the seven self-report items of the DN4 screening questionnaire. Patients were not specifically recruited for this study, as it is retrospective. Due to the retrospective nature, data on the amount of patients who did not consent was not gathered. As all patients had their data gathered in a single session, no patients were lost in follow-up studies either.

The data set which was made available had not yet been prepared for this research. The exclusion criteria for the 555 excluded participants are shown in Table 1. The table contains the steps taken to clean the data, the reasoning behind the steps, as well as the remaining patients after each step.

**Table 1.** Exclusion criteria for the participants in this study.

	Reason for removal	Amount of patients left
Step 0	Original data amount	910
Step 1	Patients in the period leading up to 2014 who had an RDC diagnosis	786
Step 2	Patients who were underage at the time of their visit to ACTA	748
Step 3	Patients who did not have a complete DN4	447
Step 4	Patients without a DC/TMD-assessment	432
Step 5	Patients who did not report any pain	355

*Note: RDC: Research Diagnostic Criteria; ACTA: Academic Centre for Dentistry Amsterdam; DN4: Douleur Neuropathique 4; DC/TMD: Diagnostic Criteria/Temporomandibular Disorders.*

All questionnaires were implemented in a comprehensive digital questionnaire that is completed by all patients before their first appointment at the clinic, as part of usual care.

At the first visit, all patients included in this research were clinically examined according to the DC/TMD protocol. In addition, the three clinical examination items of the DN4 were performed. This examination is also part of the usual care and was performed by one of the trained dentists working at the clinic. Details of the examination are provided in the subsequent paragraphs.

For this research, participants were grouped based on the presence or absence of TMD pain, myalgia, arthralgia, headache attributed to TMD and NP.

### 2.2 DC/TMD

For setting the diagnosis of temporomandibular disorders, the DC/TMD protocol [11, 22] was used. This protocol includes a standardized assessment of the history of patients’ complaints, their psychosocial status, and a clinical examination of the masticatory muscles and the temporomandibular joints [11]. The DC/TMD consists of two axes: Axis I for the assessment of a physical diagnosis based on symptoms reported by the patients and on signs present during the clinical examination; and Axis II for the assessment of psychosocial factors of importance for prognosis and treatment planning [11]. The DC/TMD is the most commonly used instrument for pain-related TMD diagnostics, with a sensitivity ≥0.86 and a specificity ≥0.98 [11]. In the present study, a distinction was made between the presence of pain-related TMD (*e.g.*, at least one DC/TMD-pain diagnosis), myalgia, arthralgia, headache attributed to TMD [23], and the absence of pain-related TMD (no DC/TMD-pain diagnosis).

### 2.3 DN4

We used the DN4 to screen the patients for NP-characteristics in the orofacial region [16]. The DN4 is a tool which consists of two parts, namely seven self-report items and three clinical examination items. In total, there are ten points to be scored, one for each item. The first seven items of the DN4 were implemented in the digital questionnaire, asking whether the patient’s pain felt like burning (1), painful cold (2), electric shocks (3), tingling (4), pins and needles (5), numbness (6) and/or itching (7). During the clinical examination, data regarding the last three items of the DN4 were collected to determine whether hypoesthesia to touch (8) and/or to prick (9) was present in the painful area, and if the pain could be caused or increased by brushing (10). This clinical data was collected by asking the patient whether the sensation of touch, pricking, or brushing was less perceived on the painful side than on the control side during the examination. The tests were performed in the intra- or extra oral region with, respectively, a cotton roll and a dental probe, as well as a small disposable composite dental brush in the intra oral region or a specifically designed brush (Somedic SENSELab, Brush no. 5, Sösdala, Sweden) for the extra oral region, according to a dedicated protocol [24]. When the patient scored positive on at least four out of ten items of the DN4, the diagnosis of possible NP was set.

### 2.4 Data analysis

To pre-screen the independent variables, Chi-square tests were used to test for the association between NP and all the categorical independent variables, including gender and the presence/absence of pain-related TMD and its subdiagnoses (*i.e.*, myalgia, arthralgia and headache attributed to TMD). For the continuous independent variable, age, an independent-sample *t*-test was used. Chi-square tests and independent-sample *t*-test were regarded as the univariate analyses. Subsequently, multivariable binary logistic regression analysis was performed on the independent variables that showed a significant correlation with NP during pre-screening, to show an association with possible NP (DN4 ≥4) as the dependent variable. The tests were done using SPSS Version 29 Software (IBM Corp., Armonk, NY, USA, 2012), with a significance threshold of α = 0.05.

## 3. Results

In total, data was collected from 355 participants (mean ± SD age = 41.9 ± 15.1 yrs; 80.6% women), all of whom reported orofacial pain complaints. Demographic data (gender and age) of all participants, the pain-related TMD group, the subgroups of pain-related TMD, the no-pain-related TMD group, the possible NP group, and the no-NP group are given in Table 2.

**Table 2.** Demographic characteristics (gender and age) of all participants, of the pain-related TMD group, the subgroups of pain-related TMD, the no-pain-related TMD group (orofacial pain without pain-related TMD), and the possible NP group (DN4 ≥4) of the 355 participants with orofacial pain.

Group			Total N	Gender	N	Min age	Max age	Mean age	SD age
All participants			355	Men	69	18	73	42.04	16.29
				Women	286	18	77	41.87	14.91
DC/TMD									
	Pain-related TMD		274	Men	43	18	71	40.47	16.58
				Women	231	18	77	41.01	14.72
		Myalgia	240	Men	41	18	71	39.02	16.33
				Women	199	18	76	41.27	14.63
		Arthralgia	171	Men	22	21	71	43.68	17.66
				Women	149	18	77	41.94	14.81
		Head attr. to TMD	73	Men	5	27	54	38.40	12.03
				Women	68	18	72	41.35	14.33
	No pain-related TMD		81	Men	26	18	73	44.65	15.59
				Women	55	18	76	45.49	15.26
NP									
	Possible NP		72	Men	16	21	73	46.19	17.34
				Women	56	18	76	43.88	12.89
	No NP		283	Men	53	18	71	46.19	17.34
				Women	230	18	77	41.39	15.34

*Note: DC/TMD: Diagnostic Criteria Temporomandibular Disorders; N: number; SD: standard deviation; Min: minimum; Max: maximum; TMD: temporomandibular disorders; Head attr.: headache attributed; NP: neuropathic pain.*

Table 3 shows the cross-tabulation information, including chi-square and *p*-value, of NP with all categorical independent variables. For the continuous variable, age, the mean and standard deviation were included for the group of participants with possible NP, as well as for the group of participants without possible NP.

**Table 3.** Characteristics of the independent variables and the dependent variable of the study, as well as the chi-square tests, representing the associated dependence between the independent variables and possible NP (DN4 ≥4) in all patients with orofacial pain.

		No NP	Possible NP	Total	Chi-square	*p*-value
Pain-related TMD		212	62	274		
No pain-related TMD		71	10	81		
	Total	283	72	355	4.09	0.043*
Myalgia						
	No	101	14	115		
	Yes	182	58	240		
	Total	283	72	355	6.92	0.009*
Arthralgia						
	No	152	32	184		
	Yes	131	40	171		
	Total	283	72	355	1.97	0.160
Head attr. to TMD						
	No	236	46	282		
	Yes	47	26	73		
	Total	283	72	355	13.37	<0.001*
Gender						
	Women	230	56	286		
	Men	53	16	69		
	Total	283	72	355	0.45	0.503
Age						
	Mean	41.28	44.39			
	Std Deviation	15.40	13.90			
	Total	283	72	355	-	0.120

Note: TMD: temporomandibular disorders; DN4: Douleur Neuropathique 4; Head attr.: headache attributed; NP: neuropathic pain; *: p < 0.05. All independent variables, except for age, were analyzed using crosstab and chi-square tests. For age, the results were analyzed using an independent-sample t-test.

From the 355 participants, 274 participants (77.2%) had pain-related TMD (one or more pain diagnoses according to the DC/TMD), whilst 81 (22.8%) had no pain-related TMD. When considering pain-related TMD subdiagnoses, there were 240 participants (67.6%) with myalgia, 171 participants (48.2%) with arthralgia, and 73 (20.6%) with headache attributed to TMD. From all 355 participants, 72 participants (20.3%) had a DN4 score ≥4, suggesting possible NP. 62 participants (86.1%) of the 72 participants with possible NP had pain-related TMD, while 10 participants with possible NP (13.9%) had no pain-related TMD (Table 3).

All ten NP components from the DN4 are shown separately in Table 4 for all participants as well as for the pain-related TMD group, the subgroups of pain-related TMD, those without pain-related TMD, and those with possible NP. The table also includes the percentage of participants within each group that have a NP component (*i.e.*, DN4 ≥4).

**Table 4.** Multivariate binary logistic regression, representing the effect of the significant variables found in the chi-square test on possible NP (DN4 ≥4) using the data of all 355 participants.

Possible NP (DN4 <4 is the reference category)		
	OR (95% CI)	*p*-value
Pain-related TMD	1.09 (0.42–2.81)	0.861
Myalgia	1.71 (0.74–3.96)	0.208
Head attr. to TMD	2.37 (1.30–4.34)	0.005*

Note: TMD: temporomandibular disorders; DN4: Douleur Neuropathique 4; Head attr.: headache attributed; NP: neuropathic pain; OR: Odds Ratio; CI: Confidence Interval; *: p < 0.05.

Out of all 274 participants with pain-related TMD, 62 (22.6%) had possible NP, for myalgia this was 58 (24.2%) out of 240, for arthralgia 40 (23.4%) out of 171, and for participants with headache attributed to TMD 26 (35.6%) out of 73. Out of the 81 participants without pain-related TMD, 10 (12.3%) had possible NP.

Using chi-square tests, possible NP was found to be significantly associated with the presence of pain-related TMD (χ^2^ = 4.088, *p* = 0.043), myalgia (χ^2^ = 6.916, *p* = 0.009) and headache attributed to TMD (χ^2^ = 13.366, *p *< 0.001). The presence of arthralgia (χ^2^ = 1.974,* p* = 0.160) and gender (χ^2^ = 0.448, *p* = 0.503) were found not to be associated with possible NP (Table 3). The patient’s age was also found not to be associated with possible NP when using an independent-sample *t*-test (*p* = 0.120).

With the chi-square test, used for pre-screening, pain-related TMD, myalgia and headache attributed to TMD were found to be significantly associated with possible NP and, as such, were used in a multivariable binary logistic regression with possible NP as the dependent variable. The analysis showed that headache attributed to TMD was significantly associated with possible NP (Odds Ratio (OR) = 2.37, 95% Confidence Interval (CI) = 1.30–4.34, *p* = 0.005), while pain-related TMD (OR = 1.088, 95% CI = 0.42–2.81, *p* = 0.861) and myalgia (OR = 1.714, 95% CI = 0.74–3.96, *p *= 0.208) were not associated with possible NP (Table 4).

In all rows of Table 5, a trend can be seen in which participants without pain-related TMD seem to have NP components less frequently than those with pain-related TMD. This trend suggests that participants without pain-related TMD, as a group, seem to experience less NP than those with pain-related TMD. In the pain-related TMD group, numbness seems to score the highest out of all NP components. Burning and tingling score very high as well, while itching and hypersensitivity to brushing seem to score relatively low for all groups. Another study found similar results for numbness, while also finding a significantly increased rate of itching and hypersensitivity to brushing between patients with possible NP and with no NP [16].

**Table 5.** NP components as included in the DN4 (number; % of positive answers) of all participants, of the pain-related TMD group, of the subgroups of pain-related TMD, and of the no-pain-related TMD group in all 355 participants with orofacial pain.

		Questionnaire							Clinical examination			
Group		Burning	Painful cold	Electric shocks	Tingling	Pins and needles	Numbness	Itching	Hypoesthesia to touch	Hypoesthesia to prick	Hypersensitivity to brushing	DN4 ≥4
All participants		94 (26.5%)	63 (17.7%)	83 (23.4%)	82 (23.1%)	88 (24.8%)	126 (35.5%)	38 (10.7%)	67 (19.0%)	66 (18.6%)	22 (6.2%)	72 (20.3%)
Pain-related TMD		74 (27.0%)	51 (18.6%)	65 (23.7%)	70 (25.5%)	69 (25.2%)	107 (39.1%)	34 (12.2%)	53 (19.3%)	58 (21.2%)	19 (6.9%)	62 (22.6%)
	Myalgia	68 (28.3%)	46 (19.2%)	58 (24.2%)	61 (25.4%)	64 (26.7%)	93 (38.2%)	33 (13.8%)	49 (20.4%)	53 (22.1%)	16 (6.7%)	58 (24.2%)
	Arthralgia	49 (28.7%)	32 (18.7%)	40 (23.4%)	46 (27.1%)	43 (25.2%)	70 (40.1%)	23 (13.5%)	35 (20.5%)	37 (21.6%)	13 (7.6%)	40 (23.4%)
	Head attr. to TMD	29 (39.7%)	19 (26.0%)	26 (35.6%)	24 (32.9%)	21 (28.8%)	36 (49.3%)	10 (13.7%)	23 (31.5%)	18 (24.7%)	7 (9.6%)	26 (35.6%)
No pain-related TMD		20 (24.7%)	12 (14.8%)	18 (22.2%)	12 (14.8%)	19 (23.5%)	19 (23.5%)	4 (4.9%)	14 (17.3%)	8 (9.9%)	3 (3.7%)	10 (12.3%)
Possible NP		50 (69.4%)	31 (43.1%)	42 (58.3%)	50 (69.4%)	44 (61.1%)	56 (77.8%)	24 (33.3%)	39 (54.2%)	34 (47.2%)	12 (16.7%)	72 (100%)
No NP		44 (15.5%)	32 (11.3%)	41 (14.5%)	32 (11.3%)	44 (15.5%)	70 (24.7%)	14 (4.9%)	28 (9.9%)	32 (11.3%)	10 (3.5%)	0 (0%)

*Note: TMD: temporomandibular disorders; DN4: Douleur Neuropathique 4; Head attr.: headache attributed; NP: neuropathic pain.*

## 4. Discussion

### 4.1 Discussion of the results

The aim of this study was to examine whether in patients with orofacial pain, an association can be found between (subdiagnoses of) pain-related TMD and possible NP. The results of the multivariate binary regression analysis (Table 4) showed that there is a significant association between headache attributed to TMD on the one hand and possible NP on the other hand. A possible explanation for this association can be the high co-morbidity between TMD and headache, as well as the neuroanatomical relationship between the anatomic areas where the TMD and headache is located [25]. Moreover, in order to set the diagnosis of headache attributed to TMD, the diagnosis of myalgia and or arthralgia should be set including familiar pain to palpation of the temporalis area. This indicates that the pain area is much more widespread compared to a diagnosis of myalgia or arthralgia alone [11]. Presence of widespread pain may involve, therefore, sensitization mechanism that are also present in NP [26, 27].

In patients with orofacial pain complaints, it can be challenging to set the proper pain diagnosis, and to distinguish between nociceptive pain and NP. It is possible that they merely occur simultaneously as comorbid conditions, but nociceptive pain and NP might also influence each other. One study found that chronic pain could be explained by either the nociceptive pain model or the NP model [21]. However, there might be a link between NP and nociceptive pain; in patients with pain-related TMD, which is considered nociceptive pain, there can be somatosensory abnormalities [28]. Due to the frequent observation of the co-existence of both nociceptive pain and NP in daily clinical practice, another study suggests assessing the presence or absence of NP components in screening for NP [29]. Understanding the presence of comorbid disease states could be helpful for characterizing and treating patients, as the complex ways TMD manifests in clinical research varies a lot [26, 30, 31]. A study on orofacial pain found that clinical signs and symptoms of nociceptive pain and NP disorders often overlap [32]. The current treatment pathways of orofacial pain may not be effective for the pain complaints and can even contribute to the chronicity of the condition [33]. A study on pain-related TMD patients showed a possible link between nociceptive pain and NP in the orofacial region [28]. This study detected somatosensory abnormalities both within and outside the primary painful region, which strongly indicates disturbances in the central processing of somatosensory stimuli [28]. One study describes how an individual, diagnosed with TMD, could present with a different neuropathic diagnosis and treatment. Even within a single diagnosis, pathophysiological mechanisms may differ between individuals and, consequently, require different personalized treatments and approaches, with respect to their individual biopsychosocial history [34].

Results from this and related studies show that not enough research has been conducted so far to investigate the possible association between nociceptive pain, like pain-related TMD, and possible NP in the orofacial region. As an association has been shown in this study between headache attributed to TMD and NP, and further studies should investigate the primary predictors for this association. The diagnostic tools for orofacial pain need to be expanded, as they still do not sufficiently clarify the possible links between NP characteristics and pain-related TMD.

### 4.2 Strengths & limitations

A strength of this study is the selection process. The entire group of adult patients with orofacial pain of the Orofacial Pain and Dysfunction Clinic of ACTA within the identified timespan was included in this study, so no selection bias was present. However, the external validity is limited by the fact that the participants were referred to a specialty clinic, meaning that the study group is not representative of the total population of orofacial pain patients.

One of the limitations of this study is that we only focussed on the mechanism of pain, viz., nociceptive and neuropathic. However, pain can also be classified in other ways, such as pain based on the etiology, *i.e.*, somatic or psychogenic, or the duration of pain, *i.e.*, whether it is acute or chronic [35]. These aspects were not considered in the present study. Using the mechanism of pain is also a strength, however, because most medical doctors base their diagnosis and treatment on pain mechanisms. A similar study in the Netherlands, with regard to lower back pain, states that the assessment of the presence and severity of a NP component is key in diagnosing chronic pain patients [36]. Although chronic pain is not necessarily a symptom but can constitute a condition in itself, the International Association for the Study of Pain (IASP) terminology does not currently reflect this understanding [37]. More studies on chronic pain as a NP component predictor could lead to a better understanding of the mechanisms of pain.

Another limitation of this study is the lack of a diagnostic tool for NP. For a patho-anatomical diagnosis of NP, sufficient knowledge is required regarding the patient’s nerve injuries, which is difficult to obtain in a clinical setting. As there is no gold standard to diagnose NP in daily practice, the highest level of certainty of NP in daily clinical care that can be accomplished is possible NP. To establish a probable diagnosis of NP, a thorough and time-consuming diagnostic examination, including qualitative and quantitative testing of the somatosensory function (QST), is needed [28]. Future studies should use these methods of QST measurements to diagnose probable NP.

### 4.3 Clinical implications

NP characteristics are present in patients with a diagnosis of pain-related TMD. Headache attributed to TMD was significantly associated with possible NP. As a subdiagnosis of pain-related TMD, this stresses the need for including a NP assessment in diagnostic protocols for pain-related TMD.

## 5. Conclusions

A significant association between pain-related TMD and possible NP in the orofacial region was found, albeit only for the subdiagnosis “headache attributed to TMD”. A more rigorous diagnosis of NP in orofacial pain patients can help in creating a better understanding of other possible associations between pain-related TMD and possible NP, as well as of the predictors for this association. As the diagnoses of pain-related TMD and NP determine the treatment pathway for a patient, with each one differing substantially from the other, further investigation of possible relationships between pain-related TMD and NP is vital to provide better care for orofacial pain patients. This study shows that there is a higher chance in pain-related TMD patients to have NP than in patients without pain-related TMD.

## References

[1] Leeuw Rd, Klasser GD. Diagnosis and management of TMD’s. In Leeuw Rd, Klasser GD (eds). Orofacial pain: guidelines for assessment, diagnosis, and management (pp.127–186). 5th edn. Quintessence Pub Co: Chicago. 2013.

[2] International Association for the Study of Pain. IASP update Fact Sheet. 2022. Available at: https://www.iasp-pain.org/wp-content/uploads/2022/10/Orofacial_Pain.pdf (Accessed: 12 May 2024).

[3] International classification of orofacial pain, 1st edition (ICOP). Cephalalgia. 2020; 40: 129–221. 10.1177/0333102419893823 32103673

[4] Proença JDS, Baad-Hansen L, Braido GVDV, Mercante FG, Campi LB, Gonçalves DADG. Lack of correlation between central sensitization inventory and psychophysical measures of central sensitization in individuals with painful temporomandibular disorder. Archives of Oral Biology. 2021; 124: 105063. 10.1016/j.archoralbio.2021.105063 33529837

[5] Dieleman JP, Kerklaan J, Huygen FJPM, Bouma PAD, Sturkenboom MCJM. Incidence rates and treatment of neuropathic pain conditions in the general population. Pain. 2008; 137: 681–688. 10.1016/j.pain.2008.03.002 18439759

[6] Macfarlane TV, Glenny A, Worthington HV. Systematic review of population-based epidemiological studies of oro-facial pain. Journal of Dentistry. 2001; 29: 451–467. 10.1016/s0300-5712(01)00041-0 11809323

[7] Okeson JP, de Leeuw R. Differential diagnosis of temporomandibular disorders and other orofacial pain disorders. Dental Clinics of North America. 2011; 55: 105–120. 10.1016/j.cden.2010.08.007 21094721

[8] Simonek M, Türp JC, Bornstein MM, Dagassan-Berndt D. Prevalence and correlation with sex, age, and dental status of bone apposition at the mandibular angle and radiographic alterations of the temporomandibular joints: a retrospective observational study in an adult Swiss population. BMC Oral Health. 2024; 24: 193. 10.1186/s12903-024-03855-0 PMC10845652 38321445

[9] Ferrillo M, Giudice A, Marotta N, Fortunato F, Di Venere D, Ammendolia A,* et al*. Pain management and rehabilitation for central sensitization in temporomandibular disorders: a comprehensive review. International Journal of Molecular Sciences. 2022; 23: 12164. 10.3390/ijms232012164 PMC9602546 36293017

[10] Lövgren A, Visscher CM, Lobbezoo F, Yekkalam N, Vallin S, Wänman A, *et al*. The association between myofascial orofacial pain with and without referral and widespread pain. Acta Odontologica Scandinavica. 2022; 80: 481–486. 10.1080/00016357.2022.2036363 35776512

[11] Schiffman E, Ohrbach R, Truelove E, Look J, Anderson G, Goulet J, *et al*. Diagnostic criteria for temporomandibular disorders (DC/TMD) for clinical and research applications: recommendations of the international RDC/TMD consortium network and orofacial pain special interest group. Journal of Oral & Facial Pain and Headache. 2014; 28: 6–27. 10.11607/jop.1151 PMC4478082 24482784

[12] Taxonomy ITFo. Part III: pain terms, a current list with definitions and notes on usage. Classification of Chronic Pain second edition (Revised). IASP: Washington, DC. 2011.

[13] Treede RD, Jensen TS, Campbell JN, Cruccu G, Dostrovsky JO, Griffin JW, *et al*. Neuropathic pain: redefinition and a grading system for clinical and research purposes. Neurology. 2008; 70: 1630–1635. 10.1212/01.wnl.0000282763.29778.59 18003941

[14] Finnerup NB, Haroutounian S, Kamerman P, Baron R, Bennett DLH, Bouhassira D,* et al*. Neuropathic pain: an updated grading system for research and clinical practice. Pain. 2016; 157: 1599–1606. 10.1097/j.pain.0000000000000492 PMC4949003 27115670

[15] Chen J, Weidner N, Puttagunta R. The impact of activity-based interventions on neuropathic pain in experimental spinal cord injury. Cells. 2022; 11: 3087. 10.3390/cells11193087 PMC9563089 36231048

[16] Bouhassira D, Attal N, Alchaar H, Boureau F, Brochet B, Bruxelle J, *et al*. Comparison of pain syndromes associated with nervous or somatic lesions and development of a new neuropathic pain diagnostic questionnaire (DN4). Pain. 2005; 114: 29–36. 10.1016/j.pain.2004.12.010 15733628

[17] Rolke R, Baron R, Maier C, Tolle TR, Treede RD, Beyer A, *et al*. Quantitative sensory testing in the German Research Network on Neuropathic Pain (DFNS): standardized protocol and reference values. Pain. 2006; 123: 231–243. 10.1016/j.pain.2006.01.041 16697110

[18] Cayrol T, van den Broeke EN, Gerard E, Meeus M, Mouraux A, Roussel N, *et al*. Chronic temporomandibular disorders are associated with higher susceptibility to develop central sensitization: a case-control study. Pain. 2023; 164: e251–e258. 10.1097/j.pain.0000000000002803 36251966

[19] Kothari SF, Baad-Hansen L, Oono Y, Svensson P. Somatosensory assessment and conditioned pain modulation in temporomandibular disorders pain patients. Pain. 2015; 156: 2545–2555. 10.1097/j.pain.0000000000000325 26307861

[20] Zafereo J, Wang‐Price S, Kandil E. Quantitative sensory testing discriminates central sensitization inventory scores in participants with chronic musculoskeletal pain: an exploratory study. Pain Practice. 2021; 21: 547–556. 10.1111/papr.12990 33342049

[21] Gatchel RJ, Peng YB, Peters ML, Fuchs PN, Turk DC. The biopsychosocial approach to chronic pain: scientific advances and future directions. Psychol Bull. 2007; 133: 581–624. 10.1037/0033-2909.133.4.581 17592957

[22] International RDC/TMD Consortium Network. DC/TMD Symptom Questionnaire (updated 05 December 2013). 2013. Available from: https://ubwp.buffalo.edu/rdc-tmdinternational/wp-content/uploads/sites/58/2017/01/DC-TMD_SQ_shortform_2013-05-12.pdf (Accessed: 12 May 2024).

[23] (IHS) HCCotIHS. Headache Classification Committee of the International Headache Society (IHS) the international classification of headache disorders, 3rd edition. Cephalalgia. 2018; 38: 1–211. 10.1177/0333102417738202 29368949

[24] Svensson P, Drangsholt M, Pfau DB, List T. Neurosensory testing of orofacial pain in the dental clinic. The Journal of the American Dental Association. 2012; 143: e37–e39. 10.14219/jada.archive.2012.0301 PMC5696631 22855909

[25] van der Meer HA, Calixtre LB, Engelbert RHH, Visscher CM, Nijhuis-van der Sanden MW, Speksnijder CM. Effects of physical therapy for temporomandibular disorders on headache pain intensity: a systematic review. Musculoskeletal Science and Practice. 2020; 50: 102277. 10.1016/j.msksp.2020.102277 33126108

[26] Vale Braido GVD, Svensson P, Dos Santos Proença J, Mercante FG, Fernandes G, de Godoi Gonçalves DA. Are central sensitization symptoms and psychosocial alterations interfering in the association between painful TMD, migraine, and headache attributed to TMD? Clinical Oral Investigations. 2023; 27: 681–690. 10.1007/s00784-022-04783-5 36383296

[27] Shrivastava M, Battaglino R, Ye L. A comprehensive review on biomarkers associated with painful temporomandibular disorders. International Journal of Oral Science. 2021; 13: 23. 10.1038/s41368-021-00129-1 PMC8322104 34326304

[28] Yang G, Baad-Hansen L, Wang K, Fu K, Xie Q, Svensson P. Somatosensory abnormalities in Chinese patients with painful temporomandibular disorders. The Journal of Headache and Pain. 2016; 17: 31. 10.1186/s10194-016-0632-y PMC4829566 27071957

[29] La Cesa S, Tamburin S, Tugnoli V, Sandrini G, Paolucci S, Lacerenza M, *et al*. How to diagnose neuropathic pain? The contribution from clinical examination, pain questionnaires and diagnostic tests. Neurological Sciences. 2015; 36: 2169–2175. 10.1007/s10072-015-2382-z 26410087

[30] Kleykamp BA, Ferguson MC, McNicol E, Bixho I, Arnold LM, Edwards RR, *et al*. The prevalence of comorbid chronic pain conditions among patients with temporomandibular disorders. The Journal of the American Dental Association. 2022; 153: 241–250.e10. 10.1016/j.adaj.2021.08.008 34952681

[31] Kleykamp BA, Ferguson MC, McNicol E, Bixho I, Arnold LM, Edwards RR, *et al*. The prevalence of psychiatric and chronic pain comorbidities in fibromyalgia: an ACTTION systematic review. Seminars in Arthritis and Rheumatism. 2021; 51: 166–174. 10.1016/j.semarthrit.2020.10.006 33383293

[32] Wang Y, Mo X, Zhang J, Fan Y, Wang K, Peter S. Quantitative sensory testing (QST) in the orofacial region of healthy Chinese: influence of site, gender and age. Acta Odontologica Scandinavica. 2018; 76: 58–63. 10.1080/00016357.2017.1383511 28958193

[33] Durham J, Breckons M, Araujo-Soares V, Exley C, Steele J, Vale L. Developing effective and efficient care pathways in chronic pain: DEEP study protocol. BMC Oral Health. 2014; 14: 6. 10.1186/1472-6831-14-6 PMC3909482 24447722

[34] Baggen JHM, Koevoets AC, Koutris M, Steegers MAH, Lobbezoo F. Chronic temporomandibular disorder pain patients with a history of neuropathic pain treatment: a narrative research on their diagnosis and treatment history. BMC Oral Health. 2024; 24: 22. 10.1186/s12903-023-03796-0 PMC10768420 38178030

[35] Baron R. Mechanisms of disease: neuropathic pain—a clinical perspective. Nature Clinical Practice Neurology. 2006; 2: 95–106. 10.1038/ncpneuro0113 16932531

[36] Timmerman H, Wilder-Smith O, van Weel C, Wolff A, Vissers K. Detecting the neuropathic pain component in the clinical setting: a study protocol for validation of screening instruments for the presence of a neuropathic pain component. BMC Neurology. 2014; 14: 94. 10.1186/1471-2377-14-94 PMC4046010 24885108

[37] Kosek E, Cohen M, Baron R, Gebhart GF, Mico J, Rice ASC,* et al*. Do we need a third mechanistic descriptor for chronic pain states? Pain. 2016; 157: 1382–1386. 10.1097/j.pain.0000000000000507 26835783

